# Detection of the First Epoxyalcohol Synthase/Allene Oxide Synthase (CYP74 Clan) in the Lancelet (*Branchiostoma belcheri*, Chordata)

**DOI:** 10.3390/ijms22094737

**Published:** 2021-04-29

**Authors:** Yana Y. Toporkova, Elena O. Smirnova, Natalia V. Lantsova, Lucia S. Mukhtarova, Alexander N. Grechkin

**Affiliations:** Kazan Institute of Biochemistry and Biophysics, FRC Kazan Scientific Center of RAS, P.O. Box 30, 420111 Kazan, Russia; yelena.smirnova@aiesec.net (E.O.S.); natamed2@yandex.ru (N.V.L.); lucia74@yandex.ru (L.S.M.)

**Keywords:** cytochrome P450, CYP74, epoxyalcohol synthase, allene oxide synthase, *Branchiostoma belcheri*

## Abstract

The CYP74 clan cytochromes (P450) are key enzymes of oxidative metabolism of polyunsaturated fatty acids in plants, some Proteobacteria, brown and green algae, and Metazoa. The CYP74 enzymes, including the allene oxide synthases (AOSs), hydroperoxide lyases, divinyl ether synthases, and epoxyalcohol synthases (EASs) transform the fatty acid hydroperoxides to bioactive oxylipins. A novel CYP74 clan enzyme CYP440A18 of the Asian (Belcher’s) lancelet (*Branchiostoma belcheri*, Chordata) was biochemically characterized in the present work. The recombinant CYP440A18 enzyme was active towards all substrates used: linoleate and α-linolenate 9- and 13-hydroperoxides, as well as with eicosatetraenoate and eicosapentaenoate 15-hydroperoxides. The enzyme specifically converted α-linolenate 13-hydroperoxide (13-HPOT) to the oxiranyl carbinol (9*Z*,11*R*,12*R*,13*S*,15*Z*)-11-hydroxy-12,13-epoxy-9,15-octadecadienoic acid (EAS product), α-ketol, 12-oxo-13-hydroxy-9,15-octadecadienoic acid (AOS product), and *cis*-12-oxo-10,15-phytodienoic acid (AOS product) at a ratio of around 35:5:1. Other hydroperoxides were converted by this enzyme to the analogous products. In contrast to other substrates, the 13-HPOT and 15-HPEPE yielded higher proportions of α-ketols, as well as the small amounts of cyclopentenones, *cis*-12-oxo-10,15-phytodienoic acid and its higher homologue, dihomo-*cis*-12-oxo-3,6,10,15-phytotetraenoic acid, respectively. Thus, the CYP440A18 enzyme exhibited dual EAS/AOS activity. The obtained results allowed us to ascribe a name “*B. belcheri* EAS/AOS” (BbEAS/AOS) to this enzyme. BbEAS/AOS is a first CYP74 clan enzyme of Chordata species possessing AOS activity.

## 1. Introduction

Lancelets or amphioxi are the only living cephalochordates [1]. These organisms, as well as the urochordates (sea squirts) and vertebrates (including the jawless lamprey and hagfish), belong to the Chordata phylum [2,3,4,5]. Despite the separate evolution of cephalochordate and vertebrates from a common ancestor (which existed more than 520 million years ago), their morphology maximally retained characteristics of vertebrate ancestor *Haikouella lanceolata* [6,7]. Lancelets are considered to be intermediate between vertebrates and invertebrates. Thereby, these organisms are widely used as a model object to study the evolution of invertebrates and the origin of vertebrates [7].

Oxylipins, the products of oxidative metabolism of polyunsaturated fatty acids, maintain homeostasis at the cellular and organismic levels in various living organisms [8]. The oxylipin biosynthesis via the lipoxygenase pathway occurs both in plants and Metazoa. Primary products of lipoxygenases, fatty acid hydroperoxides undergo further conversions, many of which are mediated by enzymes of P450 superfamily [9,10], the CYP74 clan proteins, and some other nonclassical P450s [11]. The largest diversity of CYP74s, the CYP74 family members, occur in higher plants [12,13,14]. The recent detection of CYP74s in Metazoa, Proteobacteria [15], as well as the green [16] and brown [17] algae extended the diversity of CYP74s from family to the clan. There are several distinct kinds of CYP74 enzymes, including two dehydrases, namely, allene oxide synthase (AOS) [18] and divinyl ether synthase (DES) [19], and two isomerases, namely, hydroperoxide lyase (HPL, synonym hemiacetal synthase) [20,21,22] and epoxyalcohol synthase [15,17,23,24,25]. Moreover, some CYP74 enzymes possess dual HPL/EAS or trial HPL/EAS/AOS activities [26,27,28,29].

Only a few Metazoan proteins of the CYP74 clan have been described until now. These are the ApAOS of the stony coral *Acropora palmata* [15], BfEAS (CYP440A1) of the lancelet *Branchiostoma floridae* [15], and NvEAS (CYP443D1) [23] and NvHPL/EAS (CYP443C1) [26] of the starlet sea anemone *Nematostella vectensis*. At the same time, the NCBI database contains numerous annotated but uncharacterized metazoan CYP74 clan members. Characterization of novel CYP74 clan member from lancelet species *B. belcheri* is reported in the present work. The protein exhibiting the mixed EAS/AOS activity is a first CYP74 clan enzyme of Chordata species possessing the AOS catalysis.

## 2. Results

### 2.1. Bioinformatics’ Analysis of the CYP440A18 Enzyme

The search for the putative genes of CYP74 clan in *B. belcheri* genome has been performed using TBLASTN and BLASTP tools at the NCBI database using BfEAS (CYP440A1; ACD88492.1) as query. The search revealed the target protein (458 aa; XP019641998.1) possessing 71% identity at 76% query coverage to BfEAS. Among green plants, the target protein possesses the highest identity to allene oxide synthases PpAOS2 (CYP74A8) of *Physcomitrella patens* (XP024372097.1) and LeAOS1 (CYP74A1) of *Solanum lycopersicum* (CAB88032.1): 26.95% and 26.86% identity at 83% and 75% sequence coverage, respectively. The name CYP440A18 has been assigned to this new sequence (Dr. David R. Nelson, personal communication).

**Figure 1:**
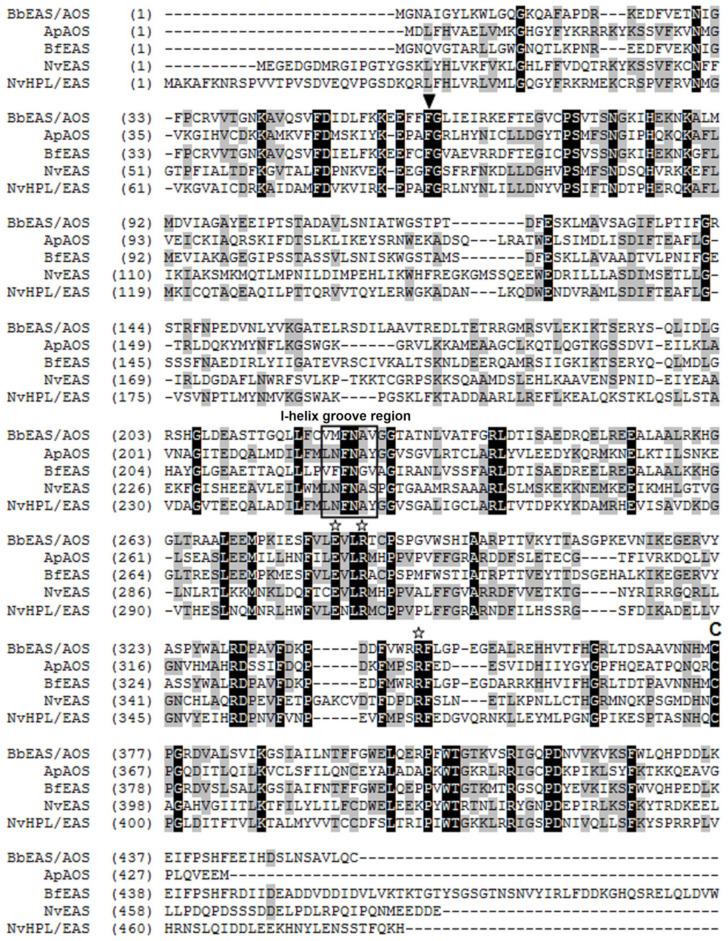
The multiple alignment of the CYP440A18 sequence with sequences of metazoan CYP74 clan members characterized earlier: Ap, *Acropora palmata* (coral); ApAOS, ACD42778.1; Bf, *Branchiostoma floridae*; BfEAS, ACD88492.1; Nv, *Nematostella vectensis*; NvEAS, XP_001636360.1; NvHPL/EAS, QJI54761.1. Conservative structures are marked as follows: the I-helix groove region is signed, while the F/L toggle, the ERR-triad, and the cysteinyl ligand are marked by triangle, stars, and C, respectively.

Alignment of the CYP440A18 enzyme with other CYP74s revealed some features of primary structure typical for CYP74s such as the I-helix groove region (earlier “hydroperoxide-binding domain” [27], SRS-4), ERR-triad, PPV-domain, nine-amino acid insertion at the heme-binding domain, and cysteinyl heme ligand (Figure 1).

**Figure 2:**
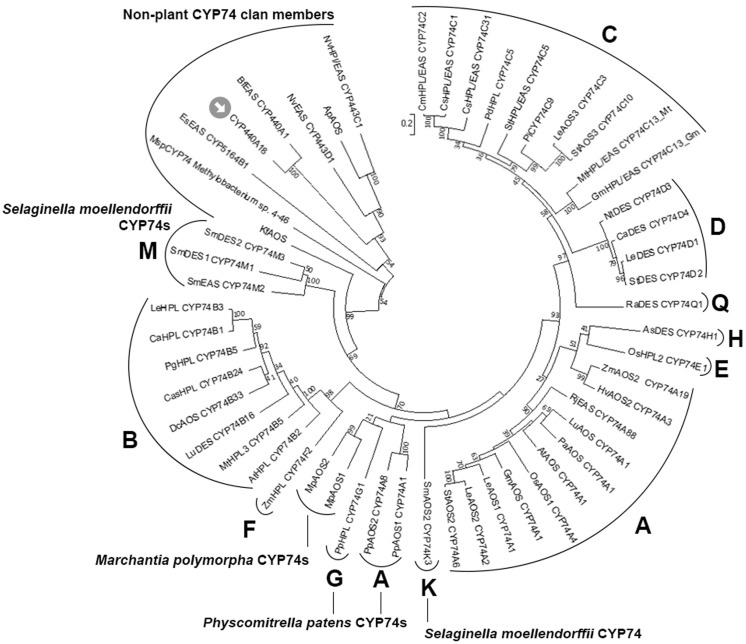
The unrooted phylogenetic tree of the CYP74 family. Classified CYP74 subfamilies are marked with their letter designations (**A**–**H**, **K**, **M**, and **Q**). The CYP440A18 enzyme is marked by an arrow. The following CYP74 clan members were used for analysis: Ap, *Acropora palmata*; ApAOS, ACD42778.1; As, *Allium sativum*; AsDES (CYP74H1), CAI30435.1; At, *Arabidopsis thaliana*; AtAOS (CYP74A1), NP199079.1; AtHPL (CYP74B2), C74B2ARATH; Bb, *Branchiostoma belcheri*; BbEAS/AOS, XP_019641998.1; Bf, *Branchiostoma floridae*; BfEAS (CYP440A1), ACD88492.1; Ca, *Capsicum annuum*; CaHPL (CYP74B1), NP001311810.1; CaDES (CYP74D4), NP001311513.1; Cas, *Camellia sinensis*; CasHPL (CYP74B24), BAU24783.1; Cs, *Cucumis sativus*; CsHPL/EAS/AOS (CYP74C31), XP004137005.1; CsHPL/EAS (CYP74C1_Cs), NP001274399.1; Cm, *Cucumis melo*; CmHPL/EAS (CYP74C2), NP001284390.1; Dc, *Daucus carota*; DcAOS (CYP74B33), XP_017248700.1; Es, *Ectocarpus siliculosus*; EsEAS (CYP5164B1), APG42673.1; Gm, *Glycine max*; GmAOS (CYP74A1), NP001236432.1; GmHPL/EAS (CYP74C13_Gm), KRH29541.1; Hv, *Hordeum vulgare*, HvAOS2 (CYP74A3), CAB86384.1; Kf, *Klebsormidium flaccidum*; KfAOS, BAS32649.1; Le, *Solanum lycopersicum*; LeAOS1 (CYP74A1), CAB88032.1; LeAOS2 (CYP74A2), AAF67141.1; LeAOS3 (CYP74C3), NP001265949.1; LeHPL (CYP74B3), AAF67142.1; LeDES (CYP74D1), NP001234527.1; Lu, *Linum usitatissimum*; LuAOS (CYP74A1), P48417.1; LuDES (CYP74B16), ADP03054.2; Mp, *Marchantia polymorpha*; MpAOS1, BAS32647.1; MpAOS2, BAS32648.1; Msp, *Methylobacterium* sp. 4-46, WP012335549.1; Mt, *Medicago truncatula*; MtHPL/EAS (CYP74C13_Mt), XP003606860.1; MtHPL3 (CYP74B4), AAY30368.1; Nt, *Nicotiana tabacum*; NtDES (CYP74D3), NP001312606.1; Nv, *Nematostella vectensis*; NvHPl/EAS (CYP443C1); NvEAS (CYP443D1), XP_001636360.1; Os, *Oryza sativa*; OsAOS1 (CYP74A4), XP015631686.1; OsHPL2 (CYP74E1), EAY85033.1; Pa, *Parthenium argentatum*; PaAOS (CYP74A1), sp|Q40778.2; Pd, *Prunus dulcis*; PdHPL (CYP74C5), CAE18065.1; Pg, *Psidium guajava*; PgHPL (CYP74B5), AAK15070.1; Pi, *Petunia inflata*; PiCYP74C9, ABC75838.1; Pp, *P. patens*; PpAOS1 (CYP74A1), XP024380613.1; PpAOS2 (CYP74A8), XP024372097.1; PpHPL (CYP74G1), CAC86920.2; Ra, *Ranunculus acris*; RaDES (CYP74Q1), AJU57209.1; Rj, *Ranunculus japonicus*; RjEAS (CYP74A88), QCR70269.1; Sm, *Selaginella moellendorffii*; SmDES1 (CYP74M1), XP002979266.1; SmDES2 (CYP74M3), XP002964012.2; SmEAS (CYP74M2), EFJ26024.1; St, *Solanum tuberosum*; StAOS2 (CYP74A6), ABD15175.1; StAOS3 (CYP74C10), CAI30876.1; StHPL/EAS (CYP74C4), XP006365486.1; StDES (CYP74D2), NP001305517.1; Zm, *Zea mays*; ZmAOS1 (CYP74A19), AAR33048.1; ZmHPL (CYP74F2), NP_001105255.2.

The catalytically essential I-helix groove region has the V-M-F-N-A-V sequence. This region contains the conserved N residue (N223 in the investigated enzyme) involved in the initial stage of CYP74 catalysis, i.e., the homolytic cleavage of the O–O bond of fatty acid hydroperoxides [15]. Phylogenetic analyses using Clustal Omega and MEGA7 (the minimum evolution and the neighbor-joining methods) confirmed that the CYP440A18 enzyme belongs to the CYP74 clan. The epoxyalcohol synthase BfEAS (CYP440A1, GenBank: ACD42778.1) of lancelet *B. floridae* is the most similar to the CYP440A18 enzyme (bootstrap support is 100) (Figure 2).

Thus, the structural and phylogenetic data confirmed that the CYP440A18 enzyme belongs to the CYP74 clan.

Alignment of open reading frame encoding the CYP440A18 enzyme with the genomic sequence mapped a gene of 5154 np length in a locus LOC109483426 (161,658–166,811, complement) and identified its structure. The coding sequence was interrupted into 5 exons by four introns. The target coding sequence adapted for expression in *E. coli* cells was custom synthesized by the Evrogen Company (Russia) and cloned into the vector pET-23a to yield the target recombinant protein with polyhistidine tag at C-terminus. His-tagged recombinant protein was obtained in BL21(DE3)pLysS strain cells (Novagen, USA) and purified by metal affinity chromatography. The enzymatic activity was controlled using the ultraviolet spectroscopy by the decrease of fatty acid hydroperoxide absorbance at 234 nm.

### 2.2. Kinetics and Substrate Specificity of the Recombinant CYP440A18 Enzyme

**Figure 3:**
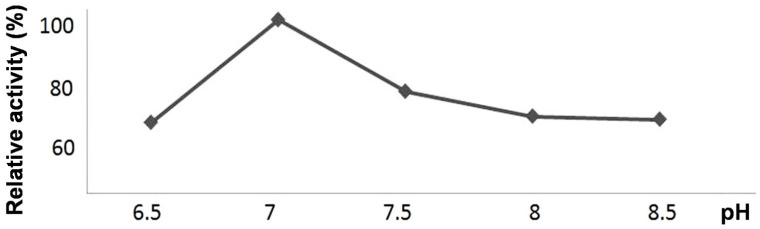
The pH dependences of catalytic activities of the recombinant CYP440A18 enzyme towards 13-HPOT.

The recombinant CYP440A18 enzyme efficiently utilized all substrates used (9-HPOD, 9-HPOT, 13-HPOD, 13-HPOT, 15-HPETE, and 15-HPEPE). The recombinant CYP440A18 enzyme exhibited the pH optimum at pH 7.0 (Figure 3).

Among C18 hydroperoxides, the CYP440A18 enzyme possessed the highest catalytic activity and affinity towards 13(*S*)-HPOT (3-5 times higher than other C_18_ hydroperoxides) (see Table 1).

As a whole, the CYP440A18 enzyme possessed higher substrate specificity towards hydroperoxides of ω3 fatty acids (α-linolenic and eicosapentaenoic acids) than hydroperoxides of ω6 fatty acids (linoleic and eicosatetraenoic acids).

### 2.3. Analysis of Products of C_18_ Hydroperoxide Conversions by the CYP440A18 Enzyme

**Figure 4:**
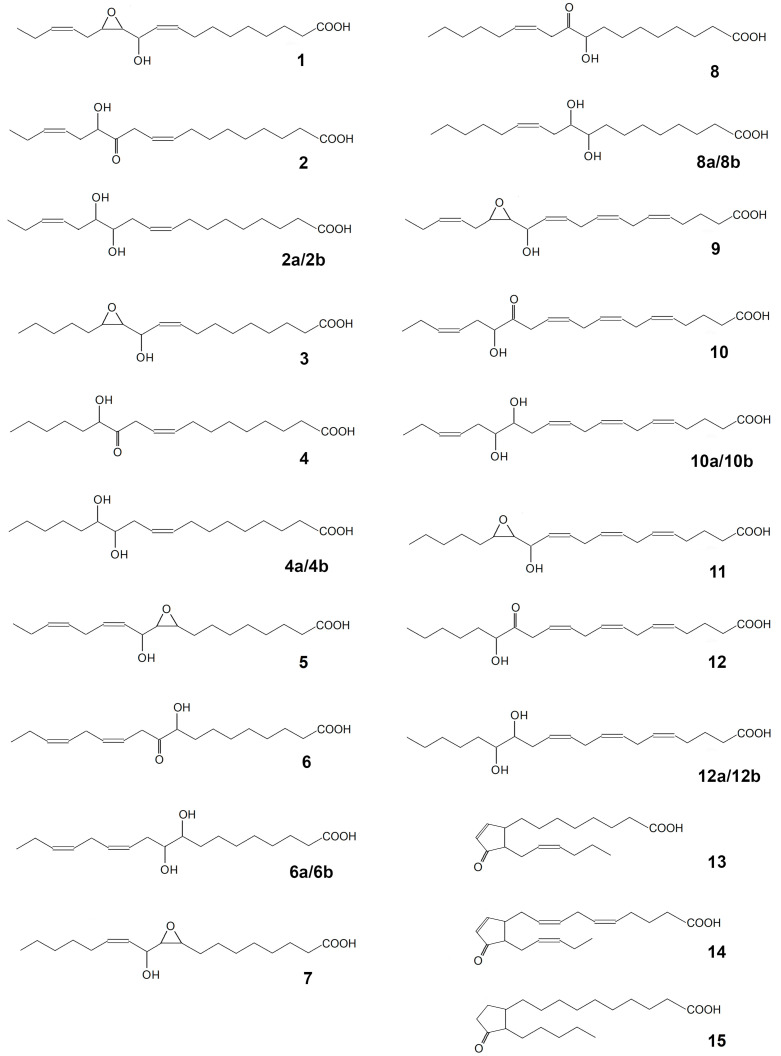
The structural formulae of products of recombinant CYP440A18 enzyme incubations with different substrates: **1**, 11-hydroxy-12,13-epoxy-9,15-octadecadienoic acid; **2**, (9*Z*,15*Z*)-12-oxo-13-hydroxy-9,15-octadecadienoic acid (α-ketol); **2a/2b**, *threo/erythro* 12,13-diols (12,13-dihydroxy-9,15-octadecadienoic acid); **3**, 11-hydroxy-12,13-epoxy-9-octadecenoic acid; 4, (9*Z*)-12-oxo-13-hydroxy-9-octadecenoic acid (α-ketol); **4a/4b**, *threo/erythro* 12,13-diols (12,13-dihydroxy-9-octadecenoic acid); **5**, 9,10-epoxy-11-hydroxy-12,15-octadecadienoic acid; **6**, (12*Z*,15*Z*)-9-hydroxy-10-oxo-12,15-octadecadienoic acid (α-ketol); **6a/6b**, *threo/erythro* 9,10-diols (9,10-dihydroxy-12,15-octadecadienoic acid); **7**, 9,10-epoxy-11-hydroxy-12-octadecenoic acid; **8**, (12*Z*)-9-hydroxy-10-oxo-12-octadecenoic acid (α-ketol); **8a/8b**, *threo/erythro* 9,10-diols (9,10-dihydroxy-12-octadecenoic acid); **9**, 13-hydroxy-14,15-epoxy-5,8,11,17-eicosatetraenoic acid; **10**, 14-oxo-15-hydroxy-5,8,11,17-eicosatetraenoic acid (α-ketol); **10a/10b**, *threo/erythro* 14,15-diols (14,15-dihydroxy-5,8,11,17-eicosatetraenoic acid); **11**, 13-hydroxy-14,15-epoxy-5,8,11-eicosatrienoic acid; **12**, 14-oxo-15-hydroxy-5,8,11-eicosatrienoic acid (α-ketol); **12a/12b**, *threo/erythro* 14,15-diols (14,15-dihydroxy-5,8,11-eicosatrienoic acid); **13**, *cis*-12-oxo-10,15-phytodienoic acid; **14**, dihomo-*cis*-12-oxo-3,6,10,15-phytodienoic acid; **15**, dihomo-12-oxophytonoic acid.

The CYP440A18 enzyme was incubated with 13-HPOT, 13-HPOD, 9-HPOT, 9-HPOD, 15-HPEPE, and 15-HPETE. The NaBH_4_-reduced (as well as the non-reduced) products (Me/TMS) of incubations were analyzed by GC–MS. The structural formulae of the identified products of the CYP440A18 enzyme are presented at the Figure 4.

**Figure 5:**
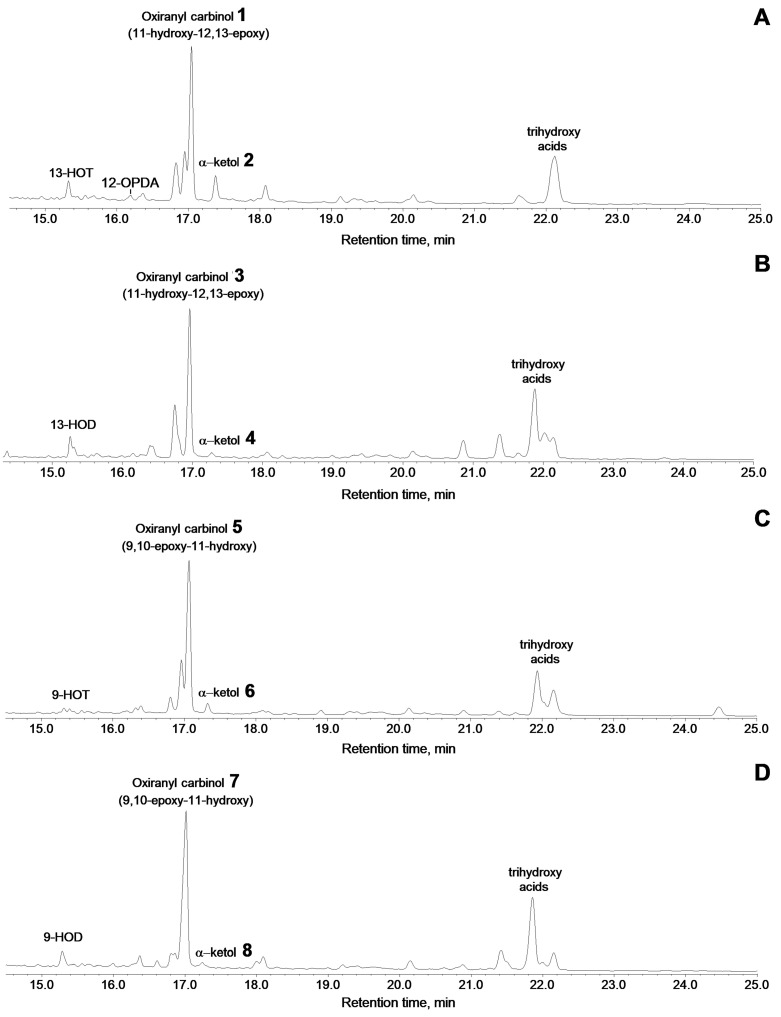
The total ion current GC–MS chromatograms of products (Me/TMS) of incubation of 13-HPOT (**A**), 13-HPOD (**B**), 9-HPOT (**C**), and 9-HPOD (**D**) with recombinant CYP440A18 enzyme. Conditions of incubation, extraction, derivatization, and analysis are described in the Materials and Methods section. The structural formulae of products are present at the Figure 4. 13-HOT, (9*Z*,11*E*,13*S*,15*Z*)-13-hydroxy-9,11,15-octadecatrienoic acid; 13-HOD, (9*Z*,11*E*,13*S*)-13-hydroxy-9,11-octadecadienoic acid; 9-HOT, (9*S*,10*E*,12*Z*,15*Z*)-9-hydroxy-10,12,15-octadecatrienoic acid; 9-HOD, (9*S*,10*E*,12*Z*)-9-hydroxy-10,12-octadecadienoic acid.

The main product of 13(*S*)-HPOT conversion by the CYP440A18 enzyme was compound **1**, the mass spectrum of which (Me/TMS, Supplementary Materials Figure S1A) possessed M^+^ at *m/z* 396 (0.1%), [M–Me]^+^ at *m/z* 381 (0.5%), [M–pentenyl]^+^ at *m/z* 327 (1%), [M–C12/C18]^+^ at *m/z* 285 (57%), *m/z* 155 (18%), *m/z* 129 (40%), and [TMS]^+^ at *m/z* 73 (100%) (Figure 5A).

The prominent fragment [M–C12/C18]^+^ at *m/z* 285 resulted from cleavage between oxirane and C11 [27]. The mass spectrum corresponded to that of (9*Z*,12*R*,13*S*,15*Z*)-11-hydroxy-12,13-epoxy-9,15-octadecadienoic acid. Catalytic hydrogenation of compound **1** over PtO_2_ followed by methylation and trimethylsilylation afforded the compound a mass spectrum corresponding to the structure of 11-hydroxy-12,13-epoxyoctadecanoic acid (Me/TMS), thus confirming the identification of product **1** as oxiranyl carbinol (9*Z*,12*R*,13*S*,15*Z*)-11-hydroxy-12,13-epoxy-9,15-octadecadienoic acid (Me/TMS).

The additional product of this reaction was the α-ketol **2** (Figure 5A, Figure S1B)—(9*Z*,15*Z*)-12-oxo-13-hydroxy-9,15-octadecadienoic acid [30,31], which upon NaBH_4_ reduction was transformed to the diastereomeric pair (*threo* and *erythro*) of 12,13-diol (12,13-dihydroxy-9,15-octadecadienoic acid, Me/TMS) **2a** and **2b**, having the identical mass spectral patterns (Figure S1C): M^+^ at *m/z* 470 (0.3%), [M–Me]^+^ at *m/z* 455 (0.6%), [M–MeOH]^+^ at *m/z* 439 (1%), [M–TMSOH–H]^+^ at *m/z* 379 (0.3%), [M–C13/C18]^+^ at *m/z* 299 (13%), [M–C1/C11]^+^ at *m/z* 273 (10%), [M–C12/C18 + TMS]^+^ at *m/z* 270 (7%), [275–TMSOH]^+^ at *m/z* 183 (18%), [M–299]^+^ at *m/z* 171 (24%), *m/z* 143 (3%), and [TMS]^+^ at *m/z* 73 (100%).

Profiles of conversions of other C_18_ hydroperoxides by the CYP440A18 enzyme looked similar to that of 13(*S*)-HPOT conversion. Thus, incubation of 13(*S*)-HPOD (Figure 5B) with the CYP440A18 enzyme resulted in formation of main product **3**, the mass spectrum of which (Me/TMS, Figure S2A) exhibited M^+^ at *m/z* 398 (0.2%), [M–Me]^+^ at *m/z* 383 (2%), [M–*n*-pentyl]^+^ at *m/z* 327 (1.5%), [M–C12/C18]^+^ at *m/z* 285 (86%), and [TMS]^+^ at *m/z* 73 (100%). The mass spectrum differed from that described above in fragment [M–Me]^+^. Catalytic hydrogenation of product **3** over PtO_2_ followed by methylation and trimethylsilylation yielded saturated analogue also described above. As a whole, the mass spectral data indicated the structure of 11-hydroxy-12,13-epoxy-9-octadecenoic acid (Me/TMS) for compound **3**. The minor product of this conversion was the α-ketol **4**, (9*Z*)-12-oxo-13-hydroxy-9-octadecenoic acid (Figure 5B and Figure S2B) [32], which upon NaBH_4_ reduction was transformed to the diastereomeric pair (*threo* and *erythro*) of 12,13-diols **4a** and **4b**, having identical mass spectral patterns (Figure S2C): M^+^ at *m/z* 472 (1.4%), [M–Me]^+^ at *m/z* 457 (2%), [M–MeOH]^+^ at *m/z* 441 (2%), [M–TMSOH–H]^+^ at *m/z* 381 (2%), [M–C13/C18]^+^ at *m/z* 299 (14%), [M–C1/C11]^+^ at *m/z* 275 (15%), [M–C12/C18 + TMS]^+^ at *m/z* 270 (7%), [275–TMSOH]^+^ at *m/z* 185 (17%), [M–299]^+^ at *m/z* 173 (35%), *m/z* 143 (10%), and [TMS]^+^ at *m/z* 73 (100%).

Conversion of 9(*S*)-HPOT by the CYP440A18 enzyme resulted in formation of compounds **5** and **6** (Figure 5C). The electron impact mass spectrum of main product **5** (Me/TMS, Figure S3A) exhibited [M–Me]^+^ at *m/z* 381 (0.3%), [M–Me–MeOH]^+^ at *m/z* 349 (0.1%), [M–TMSOH]^+^ at *m/z* 306 (1%), [M–TMSOH]^+^ at *m/z* 197 (27%), *m/z* 131 (38%), *m/z* 107 (64%), and [TMS]^+^ at *m/z* 73 (100%). Catalytic hydrogenation of product **5** over PtO_2_ followed by methylation and trimethylsilylation afforded the above-described product, the mass spectrum of which corresponded to that of 9,10-epoxy-11-hydroxyoctadecanoic acid. The data obtained enabled the identification of compound **5** as 9,10-epoxy-11-hydroxy-12,15-octadecadienoic acid. Additionally, this reaction afforded the α-ketol **6**, (12*Z*,15*Z*)-9-hydroxy-10-oxo-12,15-octadecadienoic acid (Figure 5C and Figure S3B), which upon NaBH_4_ reduction was converted to the diastereomeric pair of 9,10-diols **6a** and **6b**, possessing the identical mass spectral patterns (Figure S3C) exhibiting M^+^ at *m/z* 470 (0.7%), [M–Me]^+^ at *m/z* 455 (0.4%), [M–MeO]^+^ at *m/z* 439 (1%), [M–C11/C18]^+^ at *m/z* 361 (4%), [361–TMSOH]^+^ at *m/z* 271 (27%), [M–C10/C18]^+^ at *m/z* 259 (33%), [M–C1/C9]^+^ at *m/z* 211 (7%), *m/z* 155 (39%), *m/z* 129 (13%), *m/z* 109 (25%), [CH2 = O^+^–TMS] at *m/z* 103 (17%), and [TMS]^+^ at *m/z* 73 (100%).

The GC–MS analyses of NaBH_4_-reduced products (Me/TMS) of the CYP440A18 enzyme incubation with 9(*S*)-HPOD revealed a predominant product **7** and minor peak **8** (Figure 5D). The mass spectrum of the main product **7** (Me/TMS, Figure S4A) exhibited M^+^ at *m/z* 398 (0.2%), [M–Me]^+^ at *m/z* 383 (1%), [M–C1/C8]^+^ at *m/z* 241 (3%), [M – C1/C9]^+^ at *m/z* 212 (2%), [M–C1/C10]^+^ at *m/z* 199 (100%), and [TMS]^+^ at *m/z* 73 (100%). A prominent peak at *m/z* 199 indicated the presence of an oxiranyl carbinol function with oxirane at C9/C10 and a secondary alcohol moiety (TMS) at C11. The mass spectrum corresponded to that of 9,10-epoxy-11-hydroxy-12-octadecenoic acid (Me/TMS) [17]. Catalytic hydrogenation of product **7** over PtO_2_ followed by methylation and trimethylsilylation afforded product, mass spectrum of which indicated a structure of 9,10-epoxy-11-hydroxyoctadecanoic acid (Me/TMS). Thus, the mass spectral data substantiated the structure of 9,10-epoxy-11-hydroxy-12-octadecenoic acid for compound **7**. Additionally, this reaction afforded the α-ketol **8**, (12*Z*)-9-hydroxy-10-oxo-12-octadecenoic acid (Figure 5D and Figure S4B) [33], which upon NaBH_4_ reduction was converted to the diastereomeric pair of 9,10-diols **8a** and **8b**, possessing the identical mass spectral patterns (Figure S4C) exhibiting M^+^ at *m/z* 472 (2%), [M–Me]^+^ at *m/z* 457 (2%), [M–MeO]^+^ at *m/z* 441 (2%), [M–C11/C18]^+^ at *m/z* 361 (3%), [M–C10/C18 + TMS]^+^ at *m/z* 332 (2%), [361–TMSOH]^+^ at *m/z* 271 (12%), [M–C10/C18]^+^ at *m/z* 259 (13%), [M–C1/C9]^+^ at *m/z* 213 (9%), *m/z* 155 (25%), *m/z* 129 (32%), *m/z* 109 (30%) [CH2 = O^+^–TMS] at *m/z* 103 (16%), and [TMS]^+^ at *m/z* 73 (100%).

Among the products of the conversion of all C18 hydroperoxides studied by the CYP440A18 enzyme, trihydroxy acids, products of spontaneous hydrolysis of oxiranyl carbinols (epoxyalcohols), were also detected (Figure 5).

### 2.4. Analysis of Products of C_20_ Hydroperoxide Conversions by the CYP440A18 Enzyme

**Figure 6:**
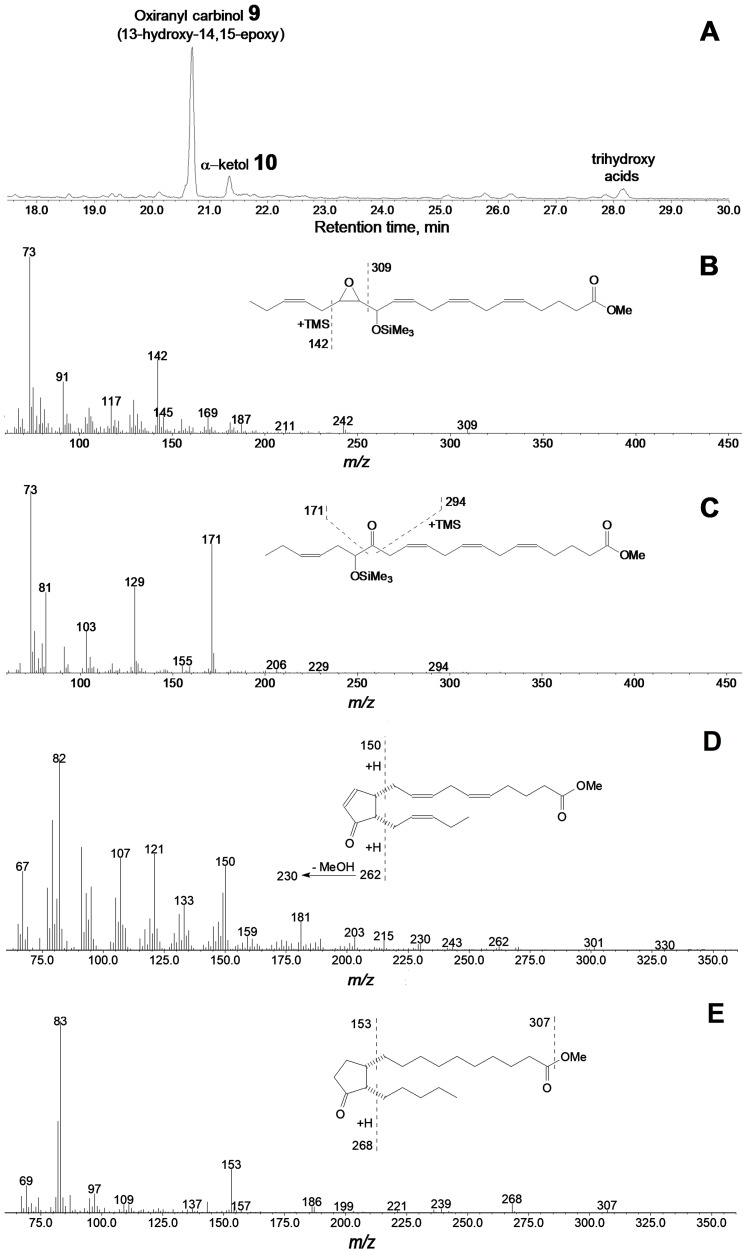
The result of GC–MS analysis of products (Me/TMS) of 15-HPEPE incubation with recombinant CYP440A18 enzyme. (**A**) The total ion current GC–MS chromatogram of products (Me/TMS) of 15-HPEPE incubation with recombinant enzyme. (**B**) The mass spectrum and fragmentation scheme (inset) for product **9**. (**C**) The mass spectrum and fragmentation scheme (inset) for product **10**. (**D**) The mass spectrum and fragmentation scheme (inset) for product **14**. (**E**) The mass spectrum and fragmentation scheme (inset) for product **15**. Conditions of incubation, extraction, derivatization, and analysis are described in the Materials and Methods section. Structures of products are presented in Figure 4.

Results of C20 hydroperoxide conversions by the CYP440A18 enzyme appeared similar to those of C18 hydroperoxide conversions. Thus, incubation of 15(*S*)-HPEPE (Figure 6A) with the CYP440A18 enzyme resulted in formation of main product **9**, the mass spectrum of which (Me/TMS, Figure 6B) exhibited M^+^ at *m/z* 420 (0.6%), [M–Me]^+^ at *m/z* 405 (0.6%), [M–C16/C20]^+^ at *m/z* 309 (2%), [M–C1/C15]^+^ at *m/z* 142 (29%), and [TMS]^+^ at *m/z* 73 (100%).

Catalytic hydrogenation of product **9** over PtO_2_ followed by methylation and trimethylsilylation yielded saturated analogue, the mass spectrum of which corresponded to that of 13-hydroxy-14,15-epoxy-eicosanoic acid (Me/TMS). As a whole, the mass spectral data indicated the structure of 13-hydroxy-14,15-epoxy-5,8,11,17-eicosatetraenoic acid (Me/TMS) for compound **9**. The minor product of this conversion was the α-ketol **10** (Figure 6C), 14-oxo-15-hydroxy-5,8,11,17-eicosatetraenoic acid (Me/TMS), which upon the NaBH_4_ reduction was transformed to the diastereomeric pair (*threo* and *erythro*) of 14,15-diols **10a** and **10b** (Figure S4D), having the identical mass spectral patterns: M^+^ at *m/z* 494 (1%), [M–Me]^+^ at *m/z* 479 (1%), [M–MeOH]^+^ at *m/z* 463 (1%), [M–C16/20]^+^ at *m/z* 425 (1.5%), [425–TMSOH]^+^ at *m/z* 335 (4%), [M–C14/C20]^+^ at *m/z* 273 (6%), [273–TMSOH]^+^ at *m/z* 183 (18%), [M–C15/C20]^+^ at *m/z* 171 (18%), and [TMS]^+^ at *m/z* 73 (94%).

**Figure 7:**
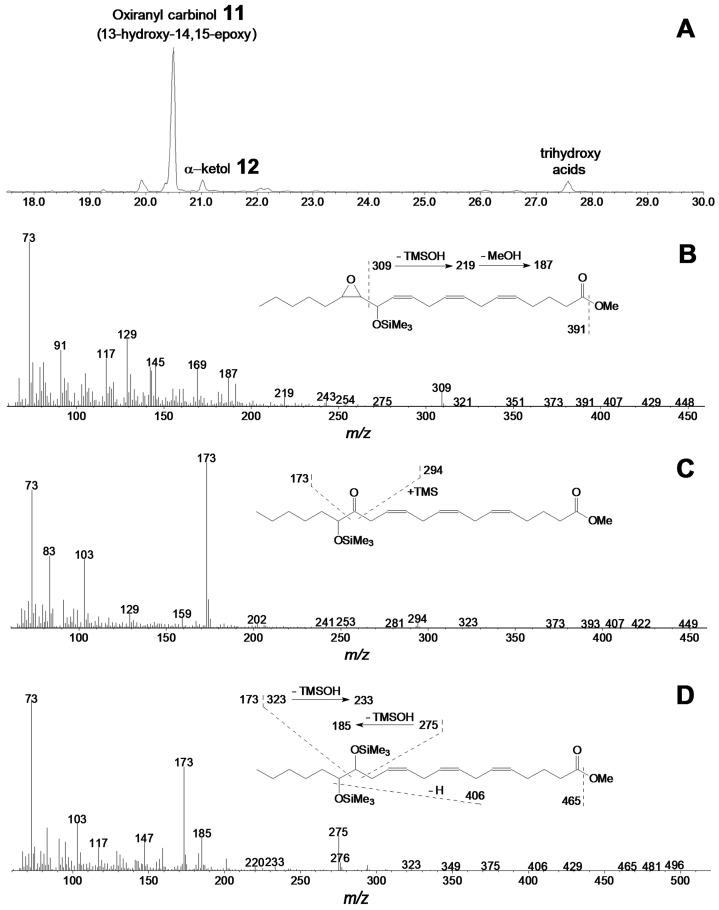
The result of GC–MS analysis of products (Me/TMS) of 15-HPETE incubation with recombinant CYP440A18 enzyme. (**A**) The total ion current GC–MS chromatogram of products (Me/TMS) of 15-HPETE incubation with recombinant enzyme. (**B**) The mass spectrum and fragmentation scheme (inset) for product **11**. (**C**) The mass spectrum and fragmentation scheme (inset) for product **12**. (**D**) The mass spectrum and fragmentation scheme (inset) for product **12a**. Conditions of incubation, extraction, derivatization, and analysis are described in the Materials and Methods section. Structures of products are presented in Figure 4.

The main product of 15(*S*)-HPETE conversion (Figure 7A) by the CYP440A18 enzyme was compound **11**, the mass spectrum of which (Me/TMS, Figure 7B) possessed M^+^ at *m/z* 422 (0.1%), [M–Me]^+^ at *m/z* 407 (0.2%), [M – C14/C20]^+^ at *m/z* 309 (7%), [309–TMSOH]^+^ at *m/z* 219 (5%), [219–MeOH]^+^ at *m/z* 187 (15%), [M–C1/C15]^+^ at *m/z* 144 (6%), and [TMS]^+^ at *m/z* 73 (100%).

The mass spectrum corresponded to that of 13-hydroxy-14,15-epoxy-8,11,17-eicosatrienoic acid (Me/TMS). Catalytic hydrogenation of compound **11** over PtO_2_ followed by methylation and trimethylsilylation afforded saturated analogue 13-hydroxy-14,15-epoxy-eicosanoic acid (Me/TMS), described above. Thus, the mass-spectral data confirmed the identification of product **11** as oxiranyl carbinol 13-hydroxy-14,15-epoxy-5,8,11-eicosatrienoic acid (Me/TMS). The minor product of this reaction was the α-ketol **12** (Figure 7C)—14-oxo-15-hydroxy-5,8,11-eicosatrienoic acid (Me/TMS), which upon NaBH_4_ reduction was transformed to the diastereomeric pair (*threo* and *erythro*) of 14,15-diol (14,15-dihydroxy-5,8,11-eicosatrienoic acid, Me/TMS) **12a** and **12b** (Figure 7D), having identical mass spectral patterns: M^+^ at *m/z* 496 (0.4%), [M–Me]^+^ at *m/z* 481 (0.3%), [M–MeO]^+^ at *m/z* 465 (0.4%), [M–TMSOH]^+^ at *m/z* 406 (0.5%), [M–C15/C20]^+^ at *m/z* 323 (1.4%), [M–C14/C20]^+^ at *m/z* 275 (20%), [323–TMSOH]^+^ at *m/z* 233 (3%), [275–TMSOH]^+^ at *m/z* 185 (20%), [M–C1/C14]^+^ at *m/z* 173 (61%), and [TMS]^+^ at *m/z* 73 (100%).

### 2.5. Formation of Cyclopentenones via Cyclization of Allene Oxides Biosynthesized from 13-HPOT and 15-HPEPE

It is noteworthy that the conversions of 13-HPOT and 15-HPEPE by CYP440A18 afforded not only α-ketols but also the *cis*-cyclopentenones **13** (Figure 5A) and **14** (Figure 6A), respectively. The mass spectrum of compound **13** (Me) possessed the following fragments: M^+^ at *m/z* 306 (7%), [M–MeO]^+^ at *m/z* 275 (12%), [M–Et–MeOH]^+^ at *m/z* 245 (13%), [M–C14/C18 + H]^+^ at *m/z* 238 (35%), [238–MeOH]^+^ at *m/z* 206 (11%), [M–C1/C6]^+^ at *m/z* 177 (18%), [M–C1/C7]^+^ at *m/z* 163 (36%), [M–C1/C8]^+^ at *m/z* 149 (31%), *m/z* 135 (26%), *m/z* 121 (39%), *m/z* 107 (74%), *m/z* 96 (99%), and *m/z* 95 (100%). The mass spectrum matched that of cyclopentenone 12-oxo-10,15-phytodienoic acid [34], the product of cyclization of allene oxide, the primary product of AOS reaction. The mass spectrum of product **14** (Me, Figure 6D) possessed the following fragments: M^+^ at *m/z* 330 (0.2%), [M–Et]^+^ at *m/z* 301 (2%), [M–MeO]^+^ at *m/z* 299 (1%), [301–MeO]^+^ at *m/z* 270 (2%), [301–MeOH]^+^ at *m/z* 269 (2%), [M–C16/C20 + H]^+^ at *m/z* 262 (2%), [262–MeOH]^+^ at *m/z* 230 (4%), *m/z* 203 (7%), [M–C11/C20]^+^ at *m/z* 181 (15 [M–C1/C10+ H]^+^ at *m/z* 150 (45%), [M–C1/C10]^+^ at *m/z* 149 (30%), *m/z* 121 (51%), *m/z* 107 (48%), *m/z* 91 (53%), and *m/z* 82 (100%). To our knowledge, no spectral data for compound **14** have been described yet, except the partial MS data reported by Ziegler et al. (1999) [35]. For further structural approval, we hydrogenated the compound **14** (Me) over PtO_2_. Hydrogenated product **15** exhibited the following mass spectral patterns (Figure 6E): M^+^ at *m/z* 338 (0.04%), [M–MeO]^+^ at *m/z* 307 (2%), [M–C16/C20 + H]^+^ at *m/z* 268 (6%), [268–MeOH]^+^ at *m/z* 236 (2%), *m/z* 187 (3%), *m/z* 187 (4%), [M–C1/C10]^+^ at *m/z* 153 (25%), *m/z* 143 (4%), and *m/z* 83 (100%), thus allowing for the identification of hydrogenation product **15** and the original compound **14** as the saturated cyclopentanone dihomo-12-oxophytonoic acid and dihomo-*cis*-12-oxo-3,6,10,15-phytotetraenoic acid (having totally four double bonds), respectively.

Ratio of different reaction products of the CYP440A18 enzyme is summarized in Table 2. The products were quantified by integration of the total ion current GC–MS chromatograms.

## 3. Discussion

The described results revealed a dual epoxyalcohol synthase/allene oxide synthase activity of the recombinant protein CYP440A18 of *B. belcheri*. On the other hand, the CYP440A18 exhibited neither HPL nor DES side activities. Thus, a name BbEAS/AOS was ascribed to the enzyme. The major products of the BbEAS/AOS were oxiranyl carbinols such as compound **1**. The same products are biosynthesized by BfEAS (CYP440A1) of the lancelet *B. floridae*, as well as NvEAS (CYP443D1) and NvHPL/EAS (CYP443C1) of the starlet sea anemone *N. vectensis* [15,23,25]. It is noteworthy that the EASs of flowering plants [17,18,20], as well as the EsEAS (CYP5164B1) of brown alga *E. siliculosus* [17], mainly produce the C10 epimer of compound **1** (with *trans*-epoxide ring). In contrast to dedicated EASs such as BfEAS (CYP440A1), NvEAS (CYP443D1), NvHPL/EAS (CYP443C1), and EsEAS (CYP5164B1), the BbEAS/AOS also possessed an AOS activity producing the considerable yields of α-ketols. Peculiarly, the hydroperoxides of ω3 fatty acids (13-HPOT and 15-HPEPE) afforded the higher proportions of AOS products upon incubations with BbEAS/AOS than all other hydroperoxides. Furthermore, the conversions of 13-HPOT and 15-HPEPE afforded the minorities of corresponding cyclopentenones, *cis*-12-oxo-10,15-phytodienoic acid and dihomo-*cis*-12-oxo-3,6,10,15-phytodienoic acid, respectively, along with α-ketols and epoxyalcohols. In contrast, α-ketols were the sole AOS products of conversions of all other tested hydroperoxides.

Allene oxides (the primary AOS products) cyclization to cyclopentenones depends on the double bond in the β,γ-position towards the oxirane [36,37]. In other words, this β,γ double bond (the ω3 double bond in allene oxides formed from 13-HPOT and 15-HPEPE) shows the effect of neighboring group participation (anchimeric assistance) elevating the cyclization rate [36,37]. The recent DFT modelling revealed that the double bond-assisted oxirane opening is the rate-limiting step of the whole conversion of allene oxide to cyclopentenone [37]. Similarly, the higher outcome of the AOS (dehydrase) products upon the BbEAS/AOS incubations with 13-HPOT and 15-HPEPE (compared to 13-HPOD and 15-HPETE) suggests the impact of ω3 double bond on the conversion of the epoxyallylic radical intermediate. Presumably, the presence of ω3 double bond facilitates the elimination of hydrogen at the oxirane to form allene oxide. Similarly, the LuDES (CYP74B16) dehydrates the 13-HPOT predominantly to divinyl ether (ω5Z)-etherolenic acid, while 13-HPOD is largely isomerized to the hemiacetal (the HPL product) [29]. These observations indicate that the ω3 double bond affects the specificity of product formation by CYP74 clan enzymes.

The BbEAS/AOS is the second CYP74 clan member found in Chordata after the BfEAS (CYP440A1) of the lancelet *Branchiostoma floridae* [15]. At the same time, it is the first enzyme of Chordata possessing AOS activity. Overall, it is the second CYP74 clan enzyme in Metazoa possessing AOS activity, after the previously described allene oxide synthase ApAOS of stony coral *A. palmata* [15]. Besides the CYP74 clan proteins, there is a distinct kind of fatty acid hydroperoxide-metabolizing detected in soft corals and cyanobacteria. These are the catalase-related haemoproteins, for instance, the AOSs of soft corals *Plexaura homomalla* [38], *Gersemia fruticosa* [39], *Capnella imbricata* [40], and *Acaryochloris marina* [41] (see [42] for a review). All these enzymes are the fusion proteins consisting of the catalase and lipoxygenase domains. Thus, the detection of BbEAS/AOS adds more intricacy to the complex picture of oxylipin biosynthesis pathways in Chordata.

### Concluding Remarks

1. The full-length coding sequence of *Branchiostoma belcheri* CYP440A18 enzyme has been expressed in *Escherichia coli* cells.
2. The recombinant CYP440A18 converted 9- and 13-hydroperoxides of linoleic and α-linolenic acids, as well as 15-hydroperoxides of eicosatetraenoic and eicosapentaenoic acids into the oxiranyl carbinols (EAS products) and α-ketols (AOS products). For example, the CYP440A18 converted the preferred substrate, 13-hydroperoxide of α-linolenic acid, into (9*Z*,11*R*,12*R*,13*S*,15*Z*)-11-hydroxy-12,13-epoxy-9,15-octadecadienoic acid (EAS product) and 12-oxo-13-hydroxy-9,15-octadecadienoic acid (AOS product). Thus, the enzyme possessed dual epoxyalcohol synthase/allene oxide synthase activity.
3. Along with α-ketols, 13-HPOT and 15-HPEPE yielded little amounts of cyclopentenones, *cis*-12-oxo-10,15-phytodienoic and dihomo-*cis*-12-oxo-3,6,10,15-phytotetraenoic acids, respectively.
4. The described BbEAS/AOS (CYP440A18) is the first epoxyalcohol synthase/allene oxide synthase (CYP74 clan) found in Chordata.

## 4. Materials and Methods

### 4.1. Materials

Linoleic, α-linolenic, eicosatetraenoic, and eicosapentaenoic acids, as well as the soybean lipoxygenase type V, were purchased from Sigma. NaBH_4_ and silylating reagents were purchased from Fluka (Buchs, Switzerland). (9*S*,10*E*,12*Z*)-9-Hydroperoxy-10,12-octadecadienoic (9-HPOD) and (9*S*,10*E*,12*Z*,15*Z*)-9-hydroperoxy-10,12,15-octadecatrienoic (9-HPOT) acids were prepared by incubation of linoleic and α-linolenic acids, respectively, with the recombinant maize 9-lipoxygenase (GeneBank: AAG61118.1) [43] at 0 °C, Na phosphate buffer (100 mM, pH 6.0), under continuous oxygen bubbling. (9*Z*,11*E*,13*S*)-13-Hydroperoxy-9,11-octadecadienoic (13-HPOD), (9*Z*,11*E*,13*S*,15*Z*)-13-hydroperoxy-9,11,15-octadecatrienoic (13-HPOT), (5*Z*,8*Z*,11*Z*,13*E*,15*S*)-13-hydroperoxy-5,8,11,13-eicosatetraenoic (15-HPETE), and (5*Z*,8*Z*,11*Z*,13*E*,15*S*,17*Z*)-13-hydroperoxy-5,8,11,13,17-eicosapentaenoic (15-HPEPE) acids were obtained by incubation of linoleic, α-linolenic, eicosatetraenoic, and eicosapentaenoic acids, respectively, with the soybean lipoxygenase type V at 23 °C, Tris-HCl buffer (50 mM, pH 9.0), under continuous oxygen bubbling. The extracted hydroperoxides (as free carboxylic acids) were purified by normal phase HPLC (NP-HPLC) on the Kromasil Si columns (7 μm; 4.0 × 250 mm; Elsico, Moscow, Russia) under the isocratic elution with the solvent mixture hexane/isopropanol/acetic acid (98.1:1.8:0.1, by volume) at a flow rate of 0.4 mL/min. Hydroperoxides were chromatographically pure and at least 98% optically pure, as judged by chiral phase HPLC [44].

### 4.2. Bioinformatic Methods

The NCBI database was used for the search of the CYP74-related genes. Primer construction and multiple sequence alignments were performed using the Vector NTI program (Invitrogen, USA). The BLAST analyses of the CYP74s were performed using the protein BLAST tool. The multiple alignments of selected CYP74 amino acid sequences and phylogenetic tree building were made with MEGA7 software [45]. Multiple alignment was performed using the Muscle method, the phylogenetic tree was built by means of the maximum likelihood method based on the Poisson correction model [46], and the bootstrap consensus tree was inferred from 1000 replicates [47]. The analysis involved 54 amino acid sequences.

### 4.3. Expression and Purification of Recombinant Enzyme

The target sequence was adapted for obtaining recombinant enzyme in *Escherichia coli* cells and synthesized in ZAO Evrogen (Russia). The resulting sequence was cloned into the pET-23a (Novagen, USA) vector using NdeI and XhoI endonucleases to yield the target recombinant protein with His-tagged C-terminus. The resulting construction was transformed into *Escherichia coli* host strain BL21(DE3)pLysS (Novagen, USA). The heterologous expression of recombinant protein was performed in the LB/M9 mixed medium (1:1, by volume) supplemented with antibiotics at 37 °C until the cell culture reached an OD_600_ of 0.6–0.8. The expression of the recombinant gene in *E. coli* cells was induced by adding 0.5 mM isopropyl-β-*D*-1-thiogalactopyranoside to the medium and the 5-aminolevulinic acid (100 mg/L), which facilitates the heme formation. The His-tagged recombinant protein was purified by immobilized metal affinity chromatography (IMAC) using Bio-Scale Mini Profinity IMAC cartridge and BioLogic LP chromatographic system (Bio-Rad, USA). The relative purity of the recombinant protein was measured by SDS-PAGE and staining of the gel with Coomassie brilliant blue R-250. Protein concentration was estimated as described before [48].

### 4.4. Kinetic Studies of Recombinant Enzyme

Enzymatic activity of the purified recombinant enzyme was determined by monitoring the decrease of the signal at 234 nm in a PB 2201 B spectrophotometer (ZAO SOLAR, Belarus) with substrate concentrations ranging from 5 to 150 μM. The analyses were performed in 0.6 mL of Na phosphate buffer (pH 6.0–8.0) at 25 °C. The initial linear regions of the kinetic curves were used to calculate the rates. The molar extinction coefficient for 9- and 13-hydroperoxides of linoleic acid at 234 nm is 25,000 M^−1^ cm^−1^. Kinetic parameters were calculated by fitting the datasets to a one-site saturation model for simple ligand binding using the SigmaPlot 11 software (Systat Software Inc., USA). Five independent experiments were performed for each specified variant.

### 4.5. Incubations of Recombinant Enzyme with Substrates

The recombinant enzyme (10 μg) was incubated with 100 μg of 9-HPOD, 9-HPOT, 13-HPOD, 13-HPOT, 15-HPETE, or 15-HPEPE in Na phosphate buffer (100 mM, 10 mL), pH 7.0, 4 °C, for 15 min. The reaction mixture was acidified to pH 6.0, and the products were extracted with hexane/ethyl acetate (1:1, by volume) mixture, methylated with ethereal diazomethane and trimethylsilylated with pyridine/hexamethyldisilazane/trimethylchlorosilane (1:1:1, by volume) mixture at 23 °C for 30 min. Then, the silylation reagents were evaporated in vacuo. The dry residue was dissolved in 100 µL of hexane and subjected to GC–MS analyses. When specified, the products were reduced with NaBH_4_, then methylated and trimethylsilylated. Alternatively, the products of NaBH_4_ reduction were hydrogenated over PtO_2_, and then methylated and trimethylsilylated. Products (without or with the preliminary NaBH_4_ reduction) were analyzed as Me esters/TMS derivatives (Me/TMS) by GC–MS, as described previously [21].

### 4.6. Methods of Instrumental Analyses

The UV spectra of the reaction mixtures were scanned during the incubations of CYP440A18 with fatty acid hydroperoxides with Varian Cary 50 spectrophotometer. Alternatively, the UV spectra of products were recorded online during the HPLC separations using an SPD-M20A diode array detector (Shimadzu, Japan). Products (Me esters or Me/TMS derivatives) were analyzed by GC–MS as described previously [21]. GC–MS analyses were performed using a Shimadzu QP5050A mass spectrometer connected to a Shimadzu GC-17A gas chromatograph equipped with an MDN-5S (5% phenyl 95% methylpolysiloxane) fused capillary column (length, 30 m; ID 0.25 mm; film thickness, 0.25 µm). Helium at a flow rate of 30 cm/s was used as the carrier gas. Injections were made in the split mode using an initial column temperature of 120 °C, injector temperature 230 °C. The column temperature was raised at 10 °C/min until it reached 240 °C. Electron impact ionization (70 eV) was used.

## Figures and Tables

**Table 1:** Kinetic parameters of the recombinant CYP440A18 enzyme towards C_18_ fatty acid hydroperoxides.

Substrate	*K_m_*, μM	*k_cat_*, s^−1^	*k_cat_/K_m_*, μM^−1^·s^–1^	Specificity, %
13-HPOT	17.6	431.8	24.5	100
13-HPOD	18.6	174.2	9.4	38.4
9-HPOT	31.8	247.9	7.8	31.8
9-HPOD	37.0	164.0	4.4	18.0

**Table 2:** Quantitation of the main products of hydroperoxide conversions by the CYP440A18 enzyme.

Substrate	α-Ketols, %	Cyclopentenones, %	Oxiranyl Carbinols, %
15-HPEPE (ω3)	17.1	tr.*	82.9
15-HPETE (ω6)	6.3	n.d.**	93.7
13-HPOT (ω3)	17.4	3.7	78.9
13-HPOD (ω6)	2.3	n.d.	97.7
9-HPOT (ω3)	5.2	n.d.	94.8
9-HPOD (ω6)	3.4	n.d.	96.6

tr.*, trace amount; n.d.**, not detected.

## Data Availability

Not applicable.

## Supplementary Materials

The following are available online at https://www.mdpi.com/article/10.3390/ijms22094737/s1.
