# Supplementary material for: Detection of the First Epoxyalcohol Synthase/Allene Oxide Synthase (CYP74 Clan) in the Lancelet (Branchiostoma belcheri, Chordata)
Source: Int J Mol Sci. 2021 Apr 29;22(9):4737. doi: 10.3390/ijms22094737 (PMC8124189; doi:10.3390/ijms22094737)
Supplement: Supplementary file 1 [file ijms-22-04737-s001.zip › ijms-1157397-supplementary.pdf]

SUPPLEMENTARY MATERIAL

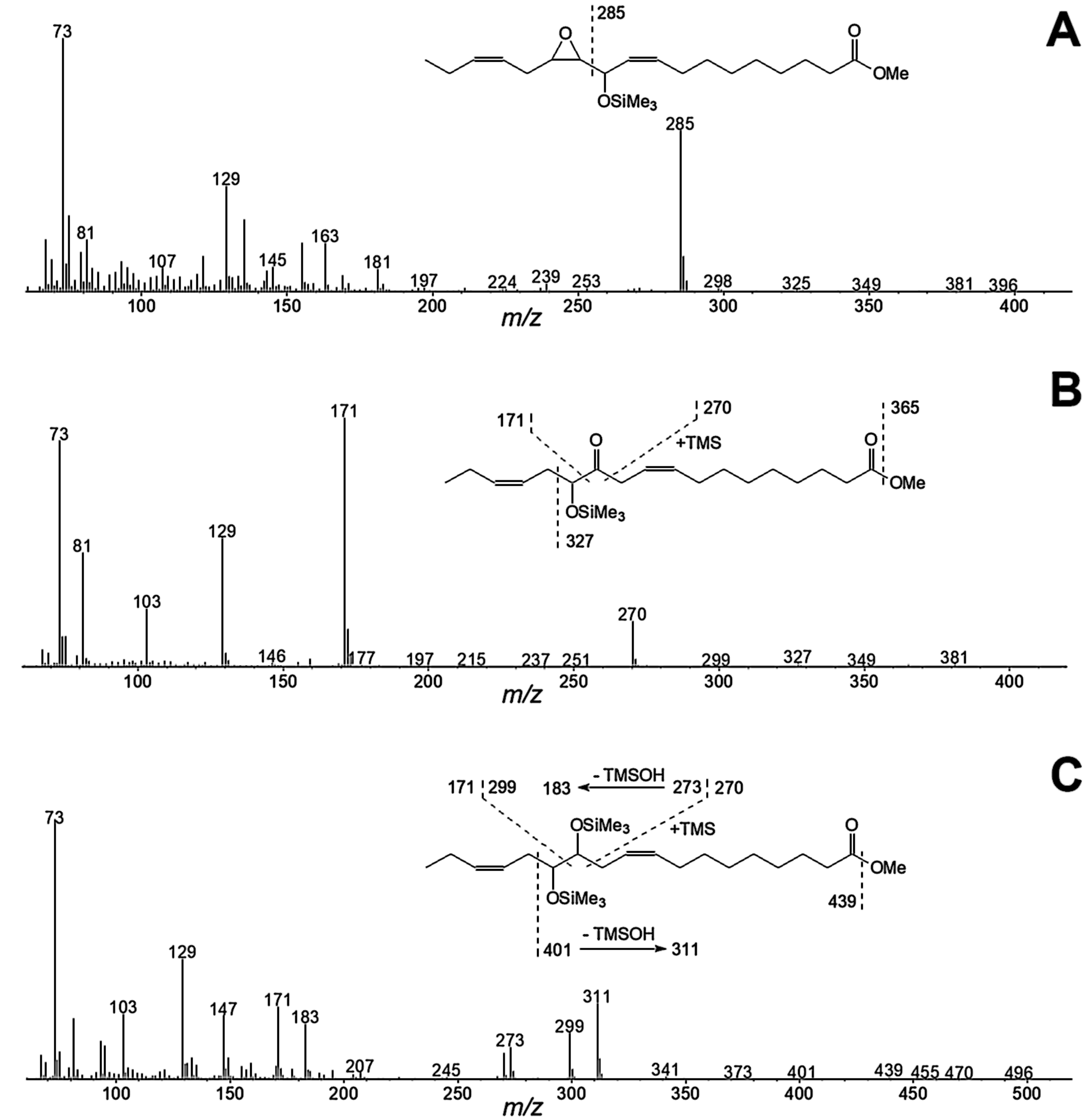

Supplementary Figure S1. The mass-spectrum and fragmentation scheme (inset) for products **1** (A), **2** (B), and **2a** (C).

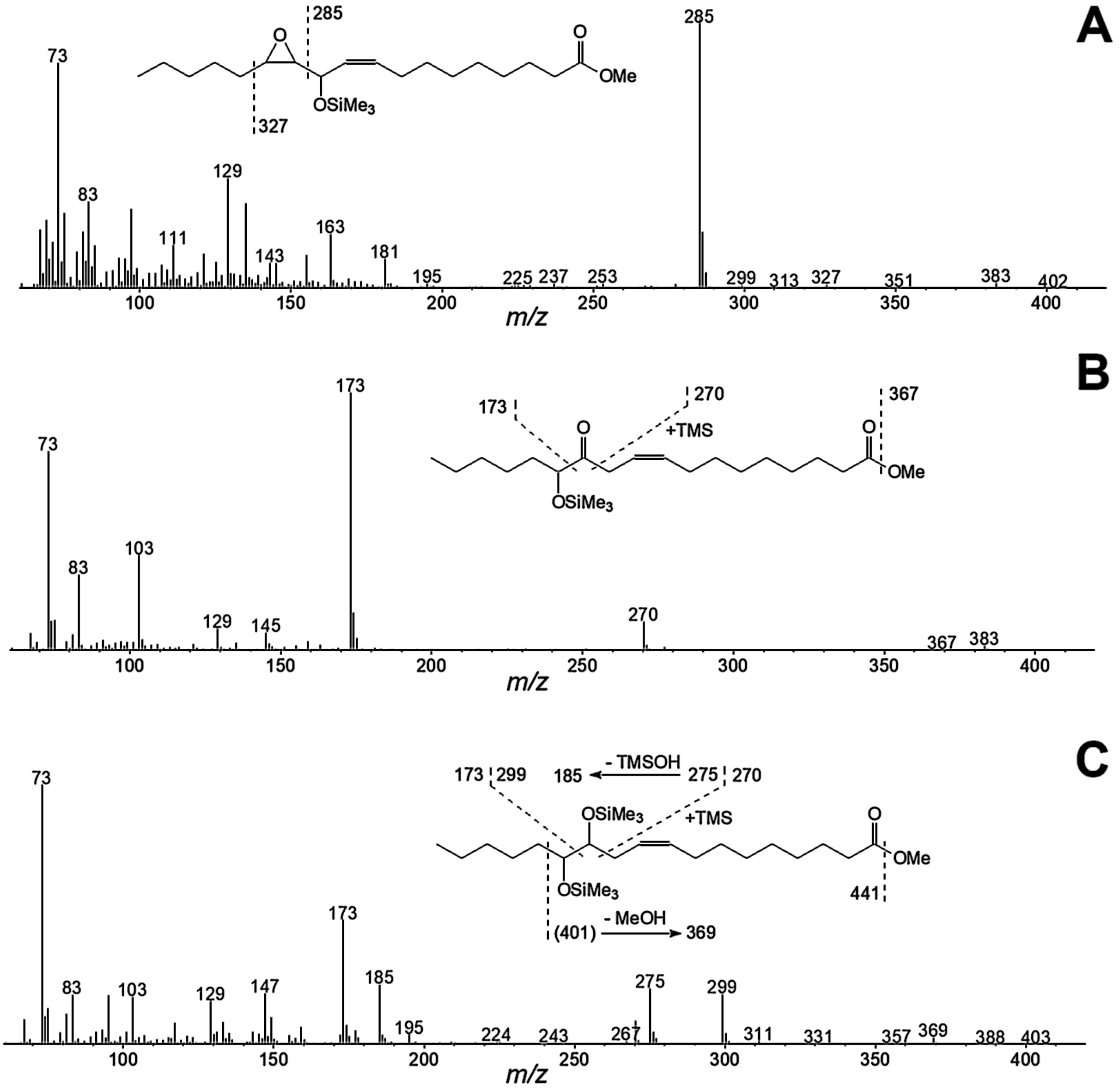

Supplementary Figure S2. The mass-spectrum and fragmentation scheme (inset) for products **3** (A), **4** (B), and **4a** (C).

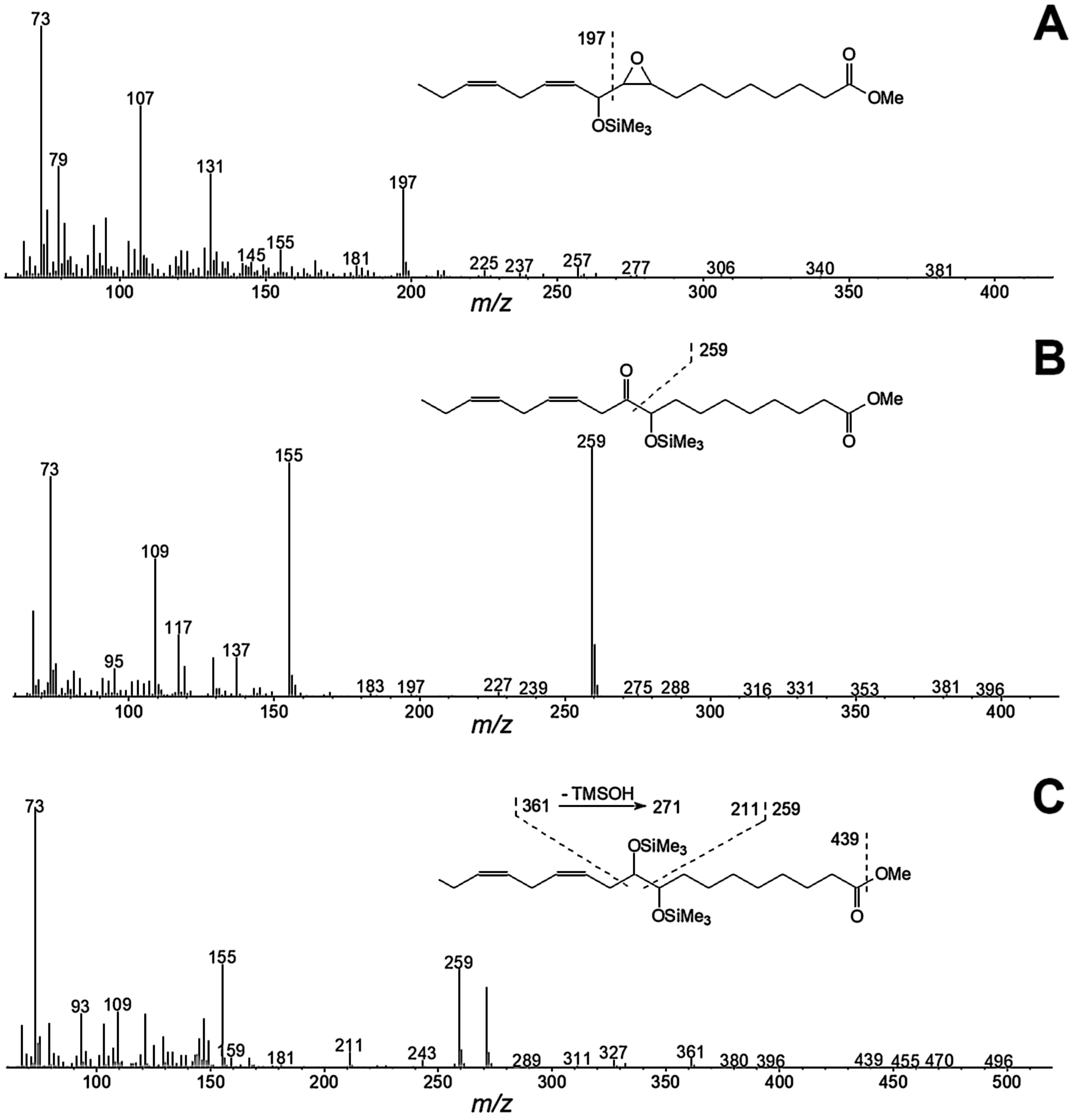

Supplementary Figure S3. The mass-spectrum and fragmentation scheme (inset) for products **5** (A), **6** (B), and **6a** (C).

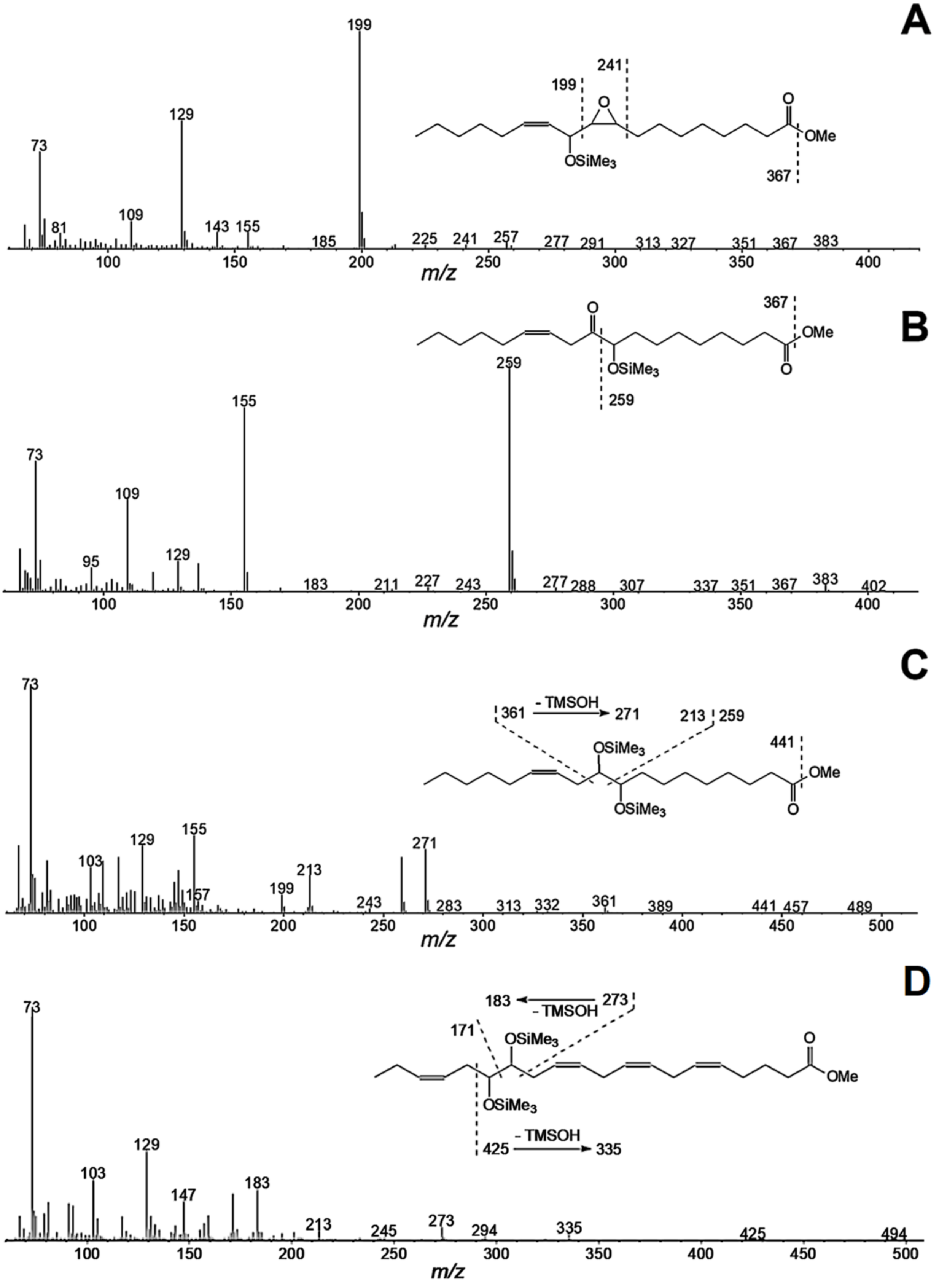

Supplementary Figure S4. The mass-spectrum and fragmentation scheme (inset) for products **7** (A), **8** (B), **8a** (C), and **10a** (D).
